# A model-based evaluation of the pharmacokinetics-pharmacodynamics (PKPD) of avibactam in combination with ceftazidime

**DOI:** 10.1093/jacamr/dlaf036

**Published:** 2025-03-11

**Authors:** Amaury O’Jeanson, Elisabet I Nielsen, Lena E Friberg

**Affiliations:** Department of Pharmacy, Uppsala University, Uppsala, Sweden; Department of Pharmacy, Uppsala University, Uppsala, Sweden; Department of Pharmacy, Uppsala University, Uppsala, Sweden

## Abstract

**Background:**

The emergence of β-lactamase-producing bacteria limits the effectiveness of β-lactam (BL) antibiotics, and the combination with a β-lactamase inhibitor (BLI) aims to counteract this resistance. However, existing guidelines primarily focus on optimizing the dosing of BLs and do not adequately address the interaction between BLs and BLIs, leading to uncertain pharmacokinetic/pharmacodynamic (PK/PD) targets and potentially suboptimal dosing strategies.

**Objectives:**

To investigate optimal PK/PD targets and dosing strategies for avibactam (BLI) combined with ceftazidime (BL) using mechanism-based PKPD models.

**Methods:**

PK models for ceftazidime and avibactam were integrated with mechanism-based PKPD models for Gram-negative bacteria. Simulations explored dose regimens in mice and humans, evaluating PK/PD indices and computing the PTA for diverse dosing strategies and infusion modes.

**Results:**

*f*AUC/MIC_CAZ/AVI_ was the most predictive index for avibactam against Enterobacteriaceae in both mice and humans, regardless of infusion mode. Against *Pseudomonas aeruginosa*, *fT* > C_T_ predicted efficacy in mice, while *f*AUC/MIC_CAZ/AVI_ and *f*Cmax/MIC_CAZ/AVI_ were more predictive in humans, particularly for continuous infusion regimens. Higher PTAs were achieved with increased avibactam doses relative to ceftazidime, particularly with 1:1 and 2:1 ceftazidime:avibactam ratios. Continuous infusion improved PTA against *P. aeruginosa* but had limited impact on Enterobacteriaceae.

**Conclusion:**

The PK/PD indices predictive of avibactam efficacy varied by species (mice and humans), bacterial strains, and mode of infusion. Dosing simulations suggest that increasing avibactam relative to ceftazidime and using continuous infusion regimens may enhance bacterial killing. These findings highlight the importance of refining dosing strategies for both components of the combination therapy.

## Introduction

The extensive use of β-lactams (BL) has led to the emergence and spread of bacterial resistance.^1^ One major type of resistance to BLs is enzyme-mediated and involves the production of β-lactamases that hydrolyse compounds containing a BL ring. To address this, combination of BLs with β-lactamase inhibitors (BLIs) have been developed. Ceftazidime/avibactam, a third-generation cephalosporin paired with an inhibitor effective against classes A, C and D β-lactamases,^2–5^ was approved in 2015 in Europe and the United States. However, optimal administration strategies for ceftazidime/avibactam remain uncertain, particularly in patient subgroups with varying treatment responses.^6,7^

Efforts to optimize the dosing strategies for ceftazidime/avibactam are therefore of continued interest. Certain administration modes, such as prolonged or continuous infusion, may improve the efficacy of BLs alone,^8^ and applying these approaches to BL/BLI combinations might similarly benefit patients. However, robust pharmacokinetic/pharmacodynamic (PK/PD) targets are needed to achieve this. Prior studies have demonstrated that PK/PD indices, often used to guide dosing, may not always translate well from mice to humans or between modes of administration,^9–11^ raising concerns about their reliability in clinical settings.

Current industry guidance for BL/BLI dosing relies on the traditional PK/PD index approach,^12^ which has limitations. PK/PD targets are typically derived from summary measures of plasma PK in rodents, making translation to humans challenging due to interspecies differences in drug disposition.^9^ Moreover, this approach usually focuses on a single timepoint (e.g. bacterial count after 24 h of drug exposure), which does not capture the time-course of drug effect or the development of resistance—an important limitation for drug combinations where dynamic interactions between the drugs occur.

For BL/BLI combinations, regulatory guidance recommends deriving the PK/PD index for the BL, and then determining an index for the BLI, with a set BL mode of administration aligning with anticipated clinical use.^12^ Based on such preclinical studies, the free drug concentration remaining above a threshold concentration for a defined portion of the dosing interval (*fT* > C_T_ with C_T_ of 1 mg/L) has previously been suggested as the best predictor of efficacy for avibactam in combination with intermittent or continuous dosing of ceftazidime.^13–15^ However, this method does not account for potential bactericidal effects of the BLI (e.g. as observed for nacubactam or avibactam),^16,17^ or synergistic interaction between the BL and BLI.^18,19^

In contrast, mechanism-based PKPD models provide a more comprehensive approach by directly linking drug concentrations to bacterial growth and killing dynamics over time, rather than relying on summary PK measures and MIC values.^20,21^ This method offers deeper insights into BL/BLI interactions, capturing dynamic bacterial responses and drug–pathogen interactions that traditional approaches often overlook. As a result, it helps optimize antibiotic therapies by addressing the limitations inherent in the PK/PD index approach.^20^

This work aimed to investigate targets and dosing strategies for avibactam used in combination with ceftazidime, employing mechanism-based PKPD models developed from *in vitro* time-kill data. The study explored the impact of experimental design, bacterial strains and study populations in the selection of the PK/PD target and the potential advantage of alternative dosing regimens, including different modes of infusion, administration frequencies and the ceftazidime/avibactam ratio.

## Materials and methods

### Overview

Previously described PK models of ceftazidime and avibactam were combined with PKPD models characterizing the effect of ceftazidime/avibactam on Gram-negative bacteria. The combined models were used to predict bacterial responses following various dose regimens in both mice and humans, with the aim to (i) investigate PK/PD targets for avibactam in combination with ceftazidime and (ii) explore optimized dosing strategies to enhance therapeutic efficacy.

### PK models

PK models from Sy *et al*.^14^ were used to predict ceftazidime and avibactam plasma PK profiles in mice. These models, developed using digitized data from an *in vivo* study involving three ceftazidime-resistant *Pseudomonas aeruginosa* isolates in murine lung and thigh infection models,^22^ were described by two-compartment models [parameter values provided in Table S1 (available as Supplementary data at *JAC-AMR* Online)].

Population PK (PopPK) models for ceftazidime and avibactam developed by Li and colleagues were used to simulate PK profiles in humans.^23^ These models were built on pooled data from phase I, II and III studies involving healthy volunteers and patients. The PK profiles were described by two-compartment models, with creatinine clearance as a key covariate explaining part of the interindividual variability in clearance. Additional covariates (age, weight, therapeutic indication, racial/regional origin, APACHE-II score) were related to clearance or central volume of distribution (parameter estimates are available in Table S2).

### PKPD models

**Figure 1.**
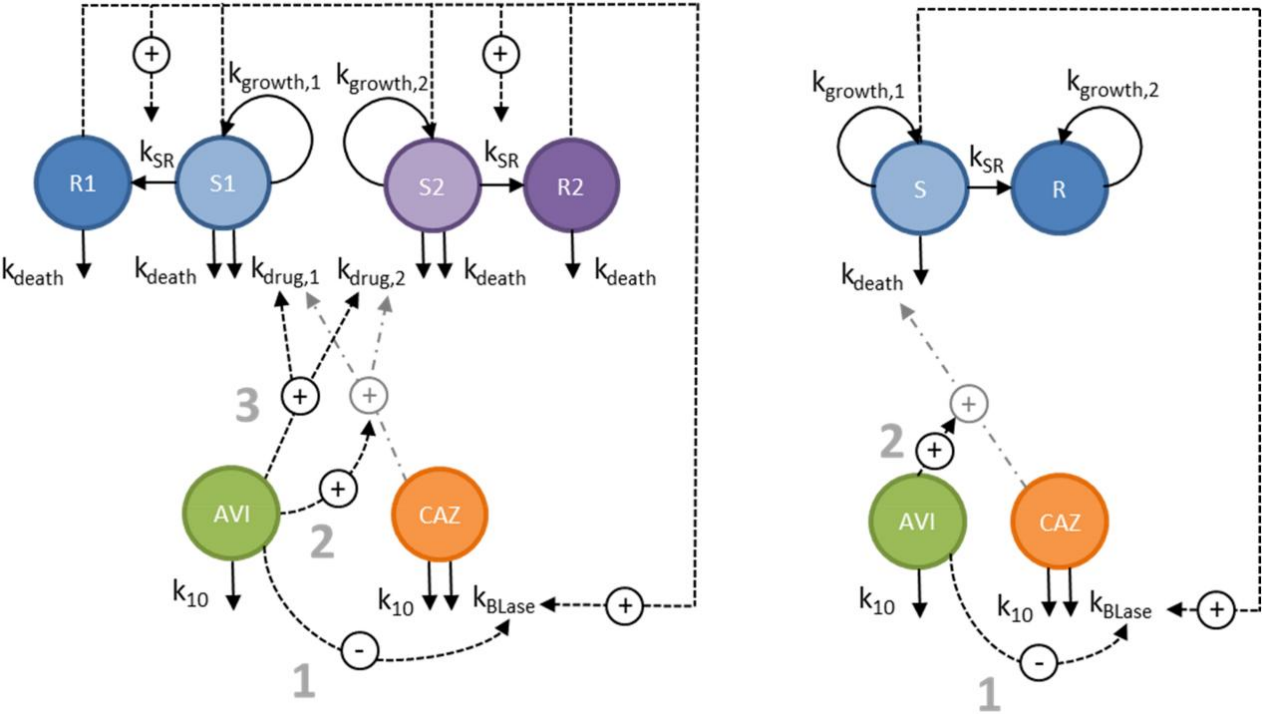
Schematic illustration of PKPD Model 1 (left)^17^ and PKPD Model 2 (right)^24^ for the interaction and effect of ceftazidime (CAZ) and avibactam (AVI). S: drug-susceptible growing state; R: drug non-susceptible resting state (in the case of PKPD Model 1: non-growing; PKPD Model 2: reduced growing); k_10_: drug elimination rate; k_BLase_: enzymatic degradation; k_death_: bacterial death rate (independent of drug in PKPD Model 1; drug-induced in PKPD Model 2); k_drug_: drug kill rate; k_SR_: transfer rate from the S to the R state; k_growth_: bacterial growth rate. The different modes of action of avibactam are numbered: (1) inhibition of β-lactamase activity; (2) potentiation of the ceftazidime effect; (3) a direct antibacterial effect. In PKPD model 1 (left), the bacterial population included two subpopulations: a main subpopulation (in blue) and a resistant subpopulation (in purple) for which susceptibility to ceftazidime and avibactam was reduced.

Two previously described mechanism-based PKPD models for the effect of ceftazidime/avibactam on Gram-negative bacteria were used in the simulations. A schematic of the model structures is shown in Figure 1, with model parameters provided in Tables S3 and S4.

‘PKPD model 1’, developed by Kristoffersson *et al*.,^17^ is based on *in vitro* static-concentration time-kill data against four strains of β-lactamase-producing ceftazidime-resistant Enterobacteriaceae: *Klebsiella pneumoniae* NCTC 13438, *K. pneumoniae* KP981690, *Escherichia coli* EO1032890 and *Enterobacter cloacae* EL871203. This model characterizes three modes of action for avibactam: inhibition of β-lactamase activity, potentiation of ceftazidime's bactericidal effect, and a direct antibacterial effect of avibactam. The model includes two bacterial subpopulations: one main (subpopulation 1) and one resistant with reduced susceptibility to ceftazidime/avibactam (subpopulation 2).

‘PKPD model 2’, developed by Sy and colleagues, is based on *in vitro* static-concentration time-kill data against three *P. aeruginosa* strains: NCTC 10783, 2154 and 9750.^24^ This model comprises of a single bacterial population and does not include a bactericidal effect of avibactam on its own.

In both PKPD models, ceftazidime degradation depended on bacterial density and was inhibited by avibactam, with rapid degradation occurring as the active bacterial population reached a high-density threshold and avibactam concentrations were low. In PKPD Model 1, an apparent delay in the onset of ceftazidime degradation was captured, while this delay was not included in PKPD Model 2. A summary of bacterial strains and MIC values for ceftazidime, avibactam and their combination is presented in Table 1.

**Table 1.** Summary of bacterial strains used for static-concentration time–kill studies of ceftazidime and avibactam from PKPD Models 1 and 2

Study	Species	Strain	β-lactamase	MIC (mg/L)		
				CAZ	AVI	CAZ/AVI^a^
PKPD Model 1 from Kristoffersson *et al.* (2020)^17^	*Klebsiella pneumoniae*	NCTC 13438	KPC-3	>64	8	4
	*Klebsiella pneumoniae*	KP981690	KPC-2	>64	16	1
	*Enterobacter cloacae*	EL871203	OXA-48	>64	32	8
	*Escherichia coli*	EO1032890	NDM-1	>64	16	512
PKPD Model 2 from Sy *et al.* (2019)^14^	*Pseudomonas aeruginosa*	NCTC 10783	AmpC	128	?^b^	16
	*Pseudomonas aeruginosa*	9750	Nr^c^	256	?^b^	32
	*Pseudomonas aeruginosa*	2154	AmpC, OXA-2	64	?^b^	4

MICs of ceftazidime alone (CAZ), avibactam alone (AVI) and CAZ/AVI combination for the strains.

^a^MICs for the combination were measured with a fixed avibactam concentration of 4 mg/L.

^b^Not measured.

^c^Not reported. *E. coli* strain EO1032890 was excluded from the simulation framework due to its high MIC value (512 mg/L) for the CAZ/AVI combination, making it clinically unsuitable for treatment with this combination.

### Simulation framework

All models were implemented in R using the mrgsolve package.^25^ Unbound plasma concentration–time profiles of ceftazidime and avibactam drove bacterial killing in PKPD models, using unbound fractions of 0.90 and 0.92 for ceftazidime and avibactam, respectively, in both mouse and human studies.^22,26,27^

### Target investigations: PK/PD index approach

Two dose-fractionation studies were simulated to investigate PK/PD index targets of avibactam in combination with ceftazidime: one in mice and one in humans. Fractionated dose regimens in both species followed a similar structure. The PK/PD index best correlating to bacterial density changes at 24 h was selected based on the coefficient of determination (r^2^).

For deriving PK/PD indices for BLIs, the European industry guideline recommends administering the BL at its intended administration mode in clinical use.^12^ In patients, the standard dose regimen for ceftazidime is 2000 mg q8h, administered as a 2-h infusion.^28^ The administered dose, dosing interval and length of infusion in patients were ‘reverse-translated’ from humans to mice, using an AUC-based approach, resulting in a derived mouse regimen of 240 mg/kg q4h as subcutaneous (SC) bolus (see Supplementary Data). This ceftazidime dose regimen was used for all bacterial strains. Various fractionated dose regimens for avibactam were simulated: total daily weight-based doses of 5, 10, 30, 50, 100, 300, 500 and 1000 mg/kg/day, administered as SC bolus in different frequencies (q2h, q4h, q6h, q8h, q12h, q24h) or as continuous infusion. A total of 56 different ceftazidime/avibactam dose regimen combinations were simulated, with a starting inoculum size of 6.0 log_10_ cfu/mL.

In humans, standard ceftazidime dose regimen (2000 mg q8h as 2 -h infusions) was paired with avibactam fractionated dose regimens of 50, 100, 150, 300, 500, 1000, 1500 and 3000 mg/day. Identical frequencies as in mice were explored (from q2h to q24h). In addition, different modes of infusion (0.5-h, 2-h, 4-h and continuous infusion) were investigated. A total of 128 ceftazidime/avibactam dose regimen combinations were simulated, including 1000 patients per regimen to account for interindividual variability. The simulated population consisted of adult patients with pneumonia (non-ventilated) and typical renal function (CrCL of 80 mL/min). The remaining covariate settings can be found in Supplementary data. The starting inoculum size was 6.0 log_10_ cfu/mL.

PK/PD indices (*fT* > C_T_, *f*C_max_/MIC_CAZ/AVI_ and *f*AUC/MIC_CAZ/AVI_) were derived for avibactam using predicted unbound concentration. For *fT* > C_T_, the threshold concentration (*C*_T_) evaluated was 1 mg/L.^15^  *f*C_max_ (highest predicted unbound concentration during the 24-h window) and *f*AUC (area under the unbound concentration–time curve over 24 h) were indexed to the MIC of the BL/BLI combination (MIC_CAZ/AVI_, determined in the presence of 4 mg/L avibactam). Relationships between PK/PD indices and avibactam efficacy were evaluated using a sigmoid Emax function:


$$E=E_{0}-\frac{PD_{max}\times X^{Hill}}{X^{Hill}+EX_{50}^{\;\;\;Hill}}$$

Where E is the PKPD model-predicted bacterial density at 24 h in log_10_ cfu/mL, E_0_ is the value without drug treatment, PD_max_ is the maximum reduction in E, X is the PK/PD index value, EX_50_ is the magnitude of X needed to achieve 50% of PD_max_ and Hill is the sigmoidal coefficient reflecting the steepness of the response curve. Curve fitting and r^2^ computation were performed in R using the nls package.^29^

### Dosing optimization: time–kill curve approach

A PTA study was simulated to investigate optimal dosing strategies for ceftazidime/avibactam in patients. The study systematically assessed the impact of three key parameters: mode of infusion, administration frequency and ceftazidime:avibactam ratio. Each parameter was evaluated independently to determine its effect on target attainment. The mode of infusion was examined by varying the infusion duration (0.5-h, 2-h, 4-h and continuous infusion) while keeping the dose constant at 2000/500 mg q8h. The impact of administration frequency was assessed by testing q6h, q8h and q12h, all administered as 2-h infusions, while ensuring a constant total daily dose of 6000/1500 mg cetazidime/avibactam. Different ceftazidime:avibactam ratios (1:1, 2:1, 3:1, 4:1, 5:1, 6:1 and 8:1) were evaluated under three fixed-dose scenarios: (i) fixed ceftazidime amount of 2000 mg, (ii) fixed avibactam amount of 500 mg and (iii) fixed total amount of ceftazidime and avibactam of 2.5 g.

Unbound plasma concentration profiles were simulated in 50 000 adult pneumonia patients with CrCL values ranging from 50 to 150 mL/min (following a normal distribution with a mean of 100 mL/min). Targets were defined as a 2-log reduction in bacterial count (cfu/mL) at 8 h, 24 h and 48 h post-administration. These simulations were performed using PopPK models from Li *et al.*,^23^ combined with PKPD models 1 or 2, to capture the dynamic relationship between drug exposure and bacterial killing, rather than relying on fixed PK/PD targets as in traditional PTA studies.

Additionally, traditional PK/PD index and time–kill curve approaches were compared. For the traditional approach, PTA for a given regimen was calculated based on the proportion of simulated patients achieving predefined PK/PD targets for a given MIC_CAZ/AVI_: 50% *fT* > MIC_CAZ/AVI_ for ceftazidime and 50% *fT* > C_T_ for avibactam, typically associated with a 2-log bacterial kill in *in vitro* and *in vivo* models.^30^ In contrast, the time-kill curve approach directly evaluated PTA by simulating bacterial density changes over time for the virtual population, and the percentage of patients achieving a 2-log kill at 24 h for a given regimen was computed.

## Results

### PK/PD indices and target investigations

**Figure 2.**
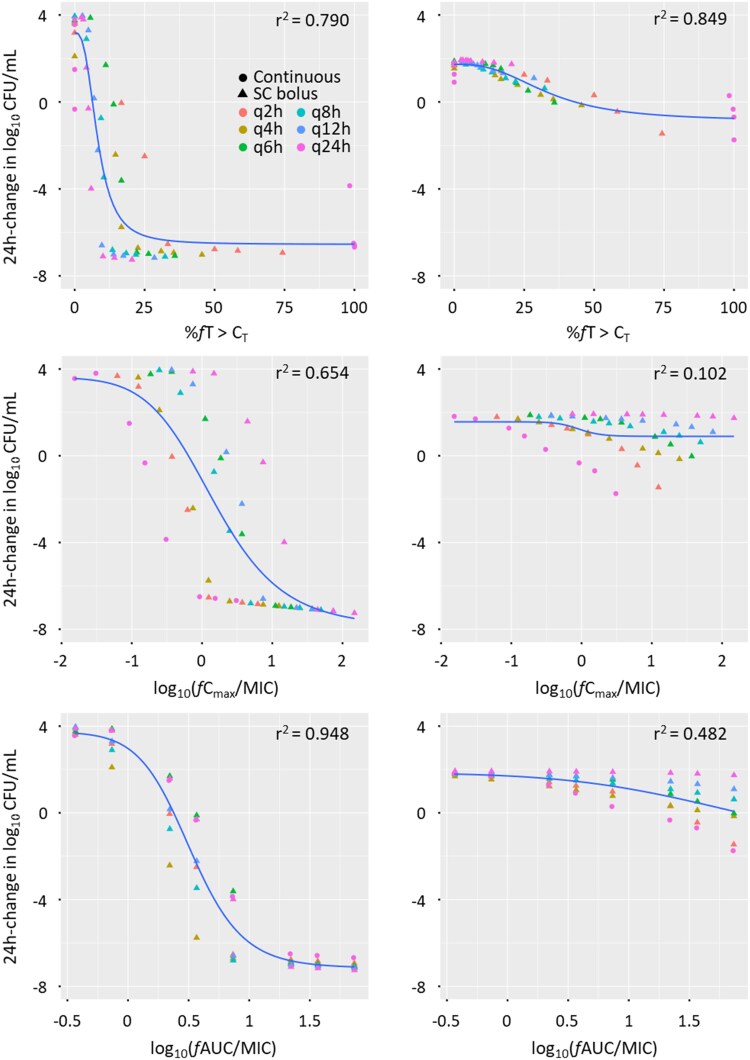
Model-predicted 24-h change in bacterial density in log_10_ cfu/mL versus the three PK/PD indices for fractionated dosing of avibactam in the presence of a fixed-dose regimen of ceftazidime (240 mg/kg q4h as SC bolus) in mice against *Klebsiella pneumoniae* NCTC 13438 (left) and *Pseudomonas aeruginosa* 2154 (right). Both strains had a MIC_CAZ/AVI_ of 4 mg/L, thus the dose fractionation design was identical (mice received a ceftazidime dose regimen of 240 mg/kg q4h as SC bolus). r^2^: coefficient of determination.

In mice, the most predictive PK/PD index of avibactam efficacy differed across bacterial strains (Table S5). Against *K. pneumoniae* (NCTC 13438, KP981690) and *E. cloacae* (EL871203), the most predictive index was *f*AUC/MIC_CAZ/AVI_. In contrast, against all three *P. aeruginosa* strains (NCTC 10783, 9750 and 2154), *fT* > C_T_ (with C_T_ of 1 mg/L) was identified as the most predictive index. Figure 2 illustrates the relationships between model-predicted changes in bacterial density over 24 h (log_10_ cfu/mL) and PK/PD indices for *K. pneumoniae* NCTC 13438 and *P. aeruginosa* 2154, both with MIC_CAZ/AVI_ 4 mg/L.

For equivalent total daily weight-based doses of avibactam in mice, larger bacterial killing at 24 h was observed against Enterobacteriaceae strains compared with *P. aeruginosa*. Against Enterobacteriaceae, the maximum bacterial killing was reached with avibactam doses of 300 mg/kg/day as further increases in dose resulted in negligible additional killing, regardless of administration frequency. Against *P. aeruginosa*, continuous or frequent intermittent infusion (q2h and q4h) generally achieved the highest bacterial killing.

In the human dose fractionation study, the predictive value of PK/PD indices for avibactam efficacy varied by bacterial strain and infusion mode. Against Enterobacteriaceae, *f*AUC/MIC_CAZ/AVI_ consistently emerged as the most predictive index in both pooled and mode-specific analyses (Table S6). Against *P. aeruginosa*, however, the optimal PK/PD index was strain- and infusion mode-dependent: *fT* > C_T_ showed superior predictive performance in the pooled analysis against strain 2154, while *f*AUC/MIC_CAZ/AVI_ performed better against strains NCTC 10783 and 9750. In mode-specific analysis, *fT* > C_T_ was most predictive for intermittent infusion (0.5-h, 2-h and 4-h infusion), whereas for continuous infusion, both *f*C_max_/MIC_CAZ/AVI_ and *f*AUC/MIC_CAZ/AVI_ showed stronger relationships to response, with similar fits and r^2^ values. For instance, against *P. aeruginosa* 9750, *fT* > C_T_ was most predictive across all infusion modes, while *f*C_max_/MIC_CAZ/AVI_ and *f*AUC/MIC_CAZ/AVI_ outperformed *fT* > C_T_ for continuous infusion.

Table 2 summarizes avibactam exposures needed to achieve bacteriostasis and 2-log bacterial kill across all studied strains in both mice and humans. In mice, against Enterobacteriaceae strains, avibactam *f*AUC/MIC_CAZ/AVI_ values of 2.19–2.38 were associated with bacteriostasis, while 3.27–3.45 achieved a 2-log kill. However, the tested regimens were insufficient to achieve adequate bacterial killing against *P. aeruginosa* strains NCTC 10783 and 9750 (MIC_CAZ/AVI_ of 16 and 32 mg/L, respectively). In contrast, in humans, avibactam *f*AUC/MIC_CAZ/AVI_ of 4.51–6.54 achieved bacteriostasis, while 12.9–22.9 achieved a 2-log kill. Specifically, against *P. aeruginosa* 2154 (MIC_CAZ/AVI_ of 4 mg/L), avibactam *fT* > C_T_ of 43.1% achieved bacteriostasis in mice, while *fT* > C_T_ of 41.6% achieved a 2-log kill in humans.

**Table 2.** Best fitting PK/PD index and magnitudes of avibactam metrics associated with bacteriostasis and bacterial killing (2-log kill) of simulated cfu in mice and humans

Species	Strain	MIC_CAZ/AVI_ (mg/L)	Mouse			Human		
			Index	Stasis	2-log kill	Index	Stasis	2-log kill
*Klebsiella pneumoniae*	KP981690	1	*f*AUC/MIC	2.19	3.27	*f*AUC/MIC	1.96	2.87
*K. pneumoniae*	NCTC 13438	4	*f*AUC/MIC	2.38	3.45	*f*AUC/MIC	1.99	2.91
*E. cloacae*	EL871203	8	fAUC/MIC	2.28	3.35	*f*AUC/MIC	1.96	2.83
*Pseudomonasaeruginosa*	2154	4	*fT* > C_T_^a^	43.1%	NaN^b^	*fT* > C_T_^a^	NaN^c^	41.6%
*P. aeruginosa*	NCTC 10783	16	*fT* > C_T_^a^	NaN^b^	NaN^b^	*f*AUC/MIC	6.54	12.9
*P. aeruginosa*	9750	32	*fT* > C_T_^a^	NaN^b^	NaN^b^	*f*AUC/MIC	4.51	22.9

By convention *f*AUC/MIC is unitless. NaN: not a number (result of an invalid calculation).

^a^Threshold concentration (C_T_) of 1 mg/L.

^b^The Emax model estimated PDmax < E0, i.e. stasis or net killing was not reached.

^c^The Emax model estimated E0 < 0 log_10_ cfu/mL.

### Dosing optimization

**Figure 3.**
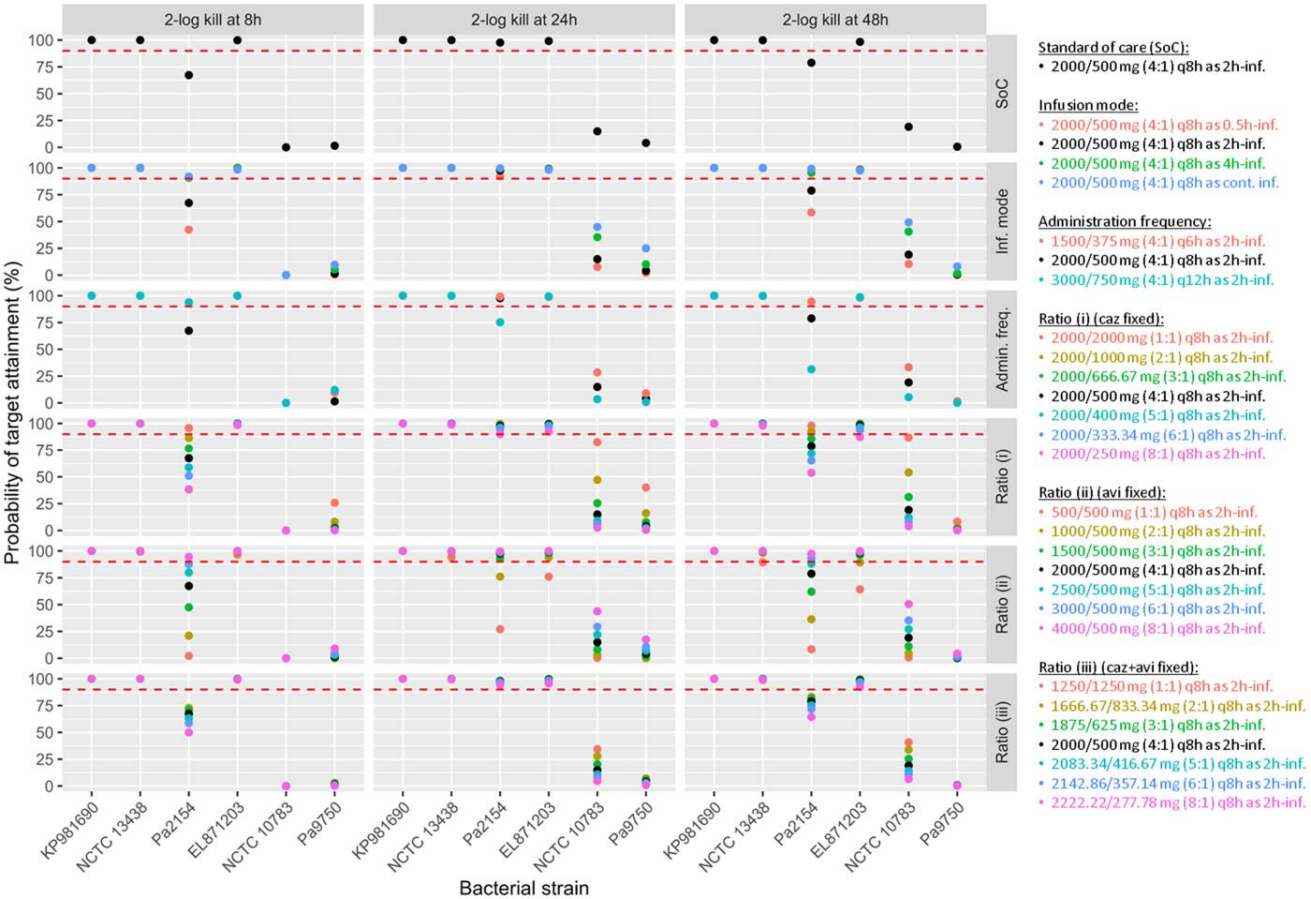
PTA for targets estimated at 2-log kill at 8 h, 24 h and 48 h. Impact of the mode of infusion, the administration frequency, and the ceftazidime/avibactam ratio. The different ceftazidime/avibactam ratios were evaluated under three fixed-dose scenarios: (i) fixed ceftazidime amount of 2000 mg, (ii) fixed avibactam amount of 500 mg and (iii) fixed total amount of ceftazidime and avibactam of 2.5 g. KP981690: *Klebsiella pneumoniae* KP981690 (MIC_CAZ/AVI_ = 1 mg/L); NCTC 13438: *K. pneumoniae* NCTC 13438 (MIC_CAZ/AVI_ = 4 mg/L); Pa2154: *Pseudomonas aeruginosa* 2154 (MIC_CAZ/AVI_ = 4 mg/L); EL871203: *E. cloacae* EL871203 (MIC_CAZ/AVI_ = 8 mg/L); NCTC 10783: *P. aeruginosa* NCTC 10783 (MIC_CAZ/AVI_ = 16 mg/L); Pa9750: *P. aeruginosa* 9750 (MIC_CAZ/AVI_ = 32 mg/L).

The PTA results from the dosing optimization investigation are presented in Figure 3. The standard of care regimen (2000/500 mg q8h as 2-h infusion) achieved satisfactory PTAs (≥90%) against all bacterial strains with MIC_CAZ/AVI_ ≤ 8 mg/L for a target of 2-log kill at 24 h. However, this regimen did not achieve satisfactory PTAs against *P. aeruginosa* strain 2154 (MIC_CAZ/AVI_ of 4 mg/L), with PTAs of 67.3%, 97.6% and 78.8% for 2-log kill at 8 h, 24 h and 48 h, respectively. Against *P. aeruginosa* strains NCTC 10783 and 9750 (MIC_CAZ/AVI_ of 16 and 32 mg/L, respectively), the standard of care did not achieve satisfactory PTAs at any PD time point.

The choice of infusion mode had limited impact on PTAs against Enterobacteriaceae strains, with differences of <3% observed between infusion modes. However, against *P. aeruginosa*, continuous infusion generally resulted in higher PTAs than intermittent infusions. For example, targeting a 2-log kill at 48 h against *P. aeruginosa* 2154, ceftazidime/avibactam 2000/500 mg q8h achieved PTAs of 58.5%, 78.8%, 95.5% and 99.2% for 0.5-h, 2-h, 4-h and continuous infusion, respectively.

The impact of administration frequency on PTAs across strains with MIC_CAZ/AVI_ ≤ 8 mg/L was minor (differences of <2%), except for *P. aeruginosa* 2154. For this strain, ceftazidime/avibactam 1500/375 mg q6h resulted in higher PTAs than 2000/500 mg q8h and 3000/750 mg q12h for all evaluated targets. Differences regarding administration frequency were also observed against *P. aeruginosa* strains NCTC 10783 and 9750, but PTAs remained unsatisfactory (<90%) for all target scenarios (2-log kill at 8 h, 24 h or 48 h).

When the avibactam proportion increased to ratios of 2:1 or 1:1 (e.g. 2000/1000 mg or 2000/2000 mg), the overall highest PTAs were achieved, suggesting that higher avibactam concentrations would improve bacterial killing.

**Figure 4.**
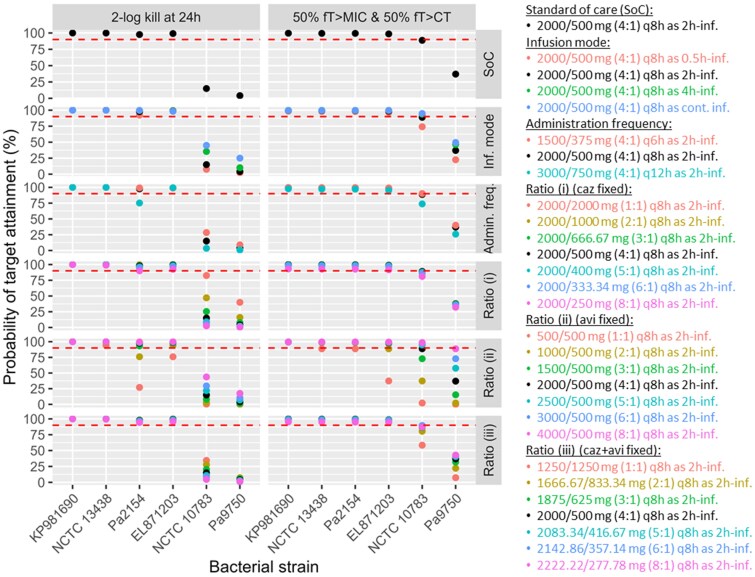
PTA results from the time–kill curve approach (2-log kill at 24 h, left) and the traditional PK/PD index approach (50% *fT* > MIC & 50% *fT* > C_T_, right). Ceftazidime/avibactam ratios were evaluated under three fixed-dose scenarios: (i) fixed ceftazidime amount of 2000 mg, (ii) fixed avibactam amount of 500 mg and (iii) fixed total amount of ceftazidime and avibactam of 2.5 g. KP981690: *Klebsiella pneumoniae* KP981690 (MIC_CAZ/AVI_ = 1 mg/L); NCTC 13438: *K. pneumoniae* NCTC 13438 (MIC_CAZ/AVI_ = 4 mg/L); Pa2154: *Pseudomonas aeruginosa* 2154 (MIC_CAZ/AVI_ = 4 mg/L); EL871203: *E. cloacae* EL871203 (MIC_CAZ/AVI_ = 8 mg/L); NCTC 10783: *P. aeruginosa* NCTC 10783 (MIC_CAZ/AVI_ = 16 mg/L); Pa9750: *P. aeruginosa* 9750 (MIC_CAZ/AVI_ = 32 mg/L).

The comparison between the traditional PK/PD index approach and the time–kill curve approach (Figure 4) revealed that PTAs for a 2-log kill at 24 h were lower than those for a joint target of 50% *fT* > MIC_CAZ/AVI_ and 50% *fT* > C_T_. This discrepancy was particularly evident against *P. aeruginosa* strain NCTC 10783 (MIC_CAZ/AVI_ of 16 mg/L). For example, with the standard of care regimen, 14.9% of patients reached 2-log kill at 24 h, whereas an 88.8% PTA was achieved for the joint target of 50% *fT* > MIC_CAZ/AVI_ and 50% *fT* > C_T_.

## Discussion

A simulation framework was employed to investigate the targets and dosing strategies of avibactam in combination with ceftazidime. *f*AUC/MIC_CAZ/AVI_ consistently emerged as the most predictive PK/PD index against Enterobacteriaceae in both mice and humans, regardless of infusion mode. In contrast, for *P. aeruginosa* the most predictive index was dependent on the explored scenario. In mice, *fT* > C_T_ was the best predictor against all three *P. aeruginosa* strains. In humans, *fT* > C_T_ was most predictive against strain 2154 in the pooled analysis, while *f*AUC/MIC_CAZ/AVI_ performed better for the other two strains. For continuous infusion, *f*AUC/MIC_CAZ/AVI_ and *f*C_max_/MIC_CAZ/AVI_ were the most predictive, while *fT* > C_T_ was generally more predictive for intermittent infusion across all *P. aeruginosa* strains. These findings suggest potential strain- and infusion mode-dependency of the PK/PD index approach.

The PK/PD simulations confirmed previously identified *fT* > C_T_ as the best index for avibactam in combination with ceftazidime against *P. aeruginosa* strains in mice.^14^ In humans, avibactam exposure required for a 2-log kill against strains NCTC 10783, 9750 and 2154 (42–100% *fT* > *C*_T_) was similar to previously reported ranges (41–100%),^14^ despite differences in dose designs. Continuous infusion data revealed that *f*AUC/MIC_CAZ/AVI_ and *f*C_max_/MIC_CAZ/AVI_ were better predictors of response, likely due to changes in the shape of the avibactam PK profiles under continuous infusion. This shift in index selection aligns with findings from Kristoffersson *et al.*^9,^ and Dhaese *et al.*^31^, highlighting how the shape of the PK profile affects the best-fitting PK/PD index.

In our dosing optimization simulations, ceftazidime/avibactam administered as continuous infusion achieved the highest PTAs against *P. aeruginosa* strains. This strategy may serve as an empirical treatment before identifying the specific bacterial strain responsible for the infection. Once identified, treatment can be tailored accordingly: continuous or prolonged infusion against *P. aeruginosa* strains, while standard 2-h infusion may work best against Enterobacteriaceae strains. However, recommendations on continuous infusion must be balanced against practical limitations, including drug degradation over time and the necessity of maintaining an automatic IV pump and an IV line for the patient.^32,33^

Increasing the avibactam dose relative to ceftazidime consistently resulted in higher PTAs across bacterial strains and time points (8 h, 24 h and 48 h). Specifically, with a fixed ceftazidime regimen and varying avibactam doses (Figure 3, scenario i), the highest PTAs were achieved with 1:1 and 2:1 ceftazidime:avibactam ratios. When allowing ceftazidime doses to vary while fixing avibactam (scenario ii), increasing the ceftazidime dose improved target attainment, but not as considerably as when increasing avibactam doses, as seen in the first scenario. Scenario iii, with a 1:1 ratio (1250/1250 mg q8h as 2 h-inf.), achieved higher target attainment than the approved 4:1 ratio. These findings suggest that increasing avibactam doses relative to ceftazidime could significantly improve bacterial killing, particularly for challenging pathogens.^34^ However, while these simulations indicate a potential strategy for optimizing dosing regimens, it is important to consider the safety profile of higher avibactam doses in clinical settings. Additionally, it is important to recognize the practical limitations of these findings, as the ceftazidime/avibactam combination is approved in a fixed 4:1 ratio, limiting the ability to adjust the ratio in practice. Therefore, while these simulations provide valuable insights, their application may be constrained by the currently approved formulation available on the market.

The model-based simulations allowed exploration of various scenarios not feasible clinically, including different dosing intervals, modes of administration, and alternative ceftazidime:avibactam ratios. This model-informed approach illustrates how integrating robust experimental data into PKPD models can improve our understanding of the interactions between BLs and BLIs to enhance drug usage. Modelling and simulation are indeed supported by regulatory authorities for efficient drug development.^35–37^ Caution should however be exercised when extrapolating beyond original dose and concentration ranges, and findings should be regarded as hypothesis-generating for new clinical studies.

The variables used for PK/PD indices of BL/BLI combinations—MIC_BL/BLI_ in %*f*T > MIC, *f*AUC/MIC or *f*Cmax/MIC, and C_T_ in %*f*T > C_T_—have inherent limitations.^38^ The MIC_BL/BLI_, as determined following EUCAST guidelines, represents the BL concentration needed at a fixed BLI concentration (4 gm/L for avibactam) rather than at the approved BL:BLI ratio used in clinical practice. This mismatch may introduce inconsistencies in PK/PD index evaluations, particularly when applied to dosing strategies. While an alternative approach could involve determining a dynamic MIC across different BL:BLI ratios,^39^ this was not possible within the scope of the current study. Furthermore, such a dynamic MIC would not be directly available for clinical dosing guidance without applying model-based approaches. The C_T_ is determined as the BLI concentration that best correlates with the bacterial response (cfu changes) in the presence of BL, rather than as a direct quantification of the level required for β-lactamase inhibition. Consequently, it is important to recognise that no single PK/PD index may be sufficient to fully describe the combined effect of BL/BLI therapy. The relationship between dosing regimens, PK profiles, β-lactamase expression and resistance mechanisms suggests that different indices may be needed depending on the clinical context and infection characteristics, emphasizing the need for a model-based approach to better integrate these factors.

While r^2^ is commonly used to evaluate model fit, its limitations, especially in non-linear models, should be recognized, as it may not accurately reflect goodness-of-fit and can lead to false conclusions.^40^ Therefore, while r^2^ was used as a measure consistent with common practice, it should be interpreted cautiously in evaluating relationships between PK/PD indices and 24-h bacterial change.

The identification of varying indices for avibactam in combination with ceftazidime across species (mice and humans), bacterial strains and modes of infusion suggests that the PK/PD approach is influenced by all these factors. This underscores the importance of comprehensive PK/PD evaluations across a range of bacterial strains during the antibiotic development process. Notably, the traditional PK/PD index approach may overestimate treatment success, particularly against *P. aeruginosa*, as it does not account for dynamic drug-bacteria interactions. This highlights the value of mechanism-based PKPD modelling, which provides a more accurate prediction of treatment outcomes by integrating these dynamics. While the choice of PK/PD target may not significantly impact clinical practice, variations in indices could pose challenges when exploring new dose regimens or addressing diverse patient populations. Therefore, understanding these variations is crucial for optimizing treatment strategies and ensuring effective outcomes in different clinical scenarios.

The applied PKPD models had been developed from *in vitro* static-concentration time–kill data without direct confirmation of their ability to predict bacterial response under dynamic conditions. However, similar PKPD models have successfully predicted dynamic time–kill outcomes for several pathogen–antibiotic combinations rather than models derived from dynamic time-kill studies, which are more suitable for capturing the impact of changing drug concentrations on bacterial response.^41–43^ Recently, Olsson *et al.* demonstrated strong agreement between *in silico* predictions using PKPD models derived from static time-kill data and experimental results from dynamic time-kill studies for an antibiotic combination (polymyxin B-minocycline), supporting the ability of such models to capture drug combination effects under changing drug concentrations. Additionally, our analysis relied on parameters estimated in earlier studies. As resistance mechanisms can impact PK/PD target estimates, additional data, including a broader range of isolate with well-characterized β-lactamase expression profiles, may further refine our findings.

In conclusion, previously developed mechanism-based PKPD models were here applied to demonstrate differences between species (mice and humans) and between bacterial strains in PK/PD indices for avibactam in combination with ceftazidime. In humans, an *f*AUC/MIC_CAZ/AVI_ ≥23 was identified as the relevant target against three Enterobacteriaceae strains and *P. aeruginosa* strains NCTC 10783 and 9750, while 42% *fT* > C_T_ was more pertinent against *P. aeruginosa* strain 2154. Additionally, the study highlighted the significant impact of mode of infusion, bacterial strains and study populations (mice or humans) on outcomes. These findings support the use of comprehensive model-based approaches that account for the full-time course of bacterial growth and killing to support selection of antibiotic dose regimens, especially for drug combinations.

## Supplementary Material

dlaf036_Supplementary_Data

## Supplementary data

Tables S1 to S6 are available as Supplementary data at *JAC-AMR* Online.
