# Supplementary material for: A model-based evaluation of the pharmacokinetics-pharmacodynamics (PKPD) of avibactam in combination with ceftazidime
Source: JAC Antimicrob Resist. 2025 Mar 11;7(2):dlaf036. doi: 10.1093/jacamr/dlaf036 (PMC11894798; doi:10.1093/jacamr/dlaf036)
Supplement: dlaf036_Supplementary_Data [file dlaf036_supplementary_data.docx]

**Supplementary data**

**Article title:** A model-based evaluation of the pharmacokinetics-pharmacodynamics (PKPD) of avibactam in combination with ceftazidime

**Short running title:** Evaluation of the PKPD for avibactam

**Author names:** Amaury O’Jeanson, Elisabet I. Nielsen, Lena E. Friberg

**Affiliation:** Department of Pharmacy, Uppsala University, Uppsala, Sweden

**Table S1.** Parameter estimates for PK models of ceftazidime/avibactam (CAZ/AVI) in mouse^1^

| Parameter | Unit | Description | Estimate (RSE%) | |
| --- | --- | --- | --- | --- |
|  |  |  | CAZ | AVI |
| CL | L/h/kg | Systemic clearance | 1.74 (12.3) | 2.98 (2.0) |
| V1 | L/kg | Volume of central compartment | 0.633 (11.3) | 0.819 (1.5) |
| Q | L/h/kg | Inter-compartmental clearance | 0.154 (69.5) | 0.174 (2.7) |
| V2 | L/kg | Volume of peripheral compartment | 0.183 (53.2) | 0.36 (8.3) |
| Q_t_ | L/h/kg | Flow rate from blood compartment to lung compartment | 0.275 (11.1) | 0.137 (13.1) |
| V3 | L/kg | Volume of lung compartment | 0.235 (8.3) | 0.226 (14.1) |
| K_p_ | - | Partition coefficient | 0.2 FIXED | 0.25 FIXED |
| σ_1_ | - | Proportional error for plasma data | 0.197 (8.5) | 0.0895 (10.6) |
| σ_2_ | - | Proportional error for ELF data | 2.33 (54.1) | 0.961 (64.9) |

**Table S2.** Parameter estimates for PopPK models of CAZ/AVI in human^2^

| Parameter | Unit | Description | Estimate (RSE%) | |
| --- | --- | --- | --- | --- |
|  |  |  | CAZ | AVI |
| CL | L/h | Systemic clearance | 6.95 (1.7) | 10.2 (1.8) |
| V1 | L | Volume of central compartment | 10.5 (13.1) | 11.1 (9.9) |
| Q | L/h | Inter-compartmental clearance | 31.5 (18.8) | 5.44 (13.9) |
| V2 | L | Volume of peripheral compartment | 7.57 (9.0) | 6.91 (6.5) |
| Slope1_CRCL_CL | - | If CrCL < 100 mL/min: Slope1∙CrCL | 0.0103 (0.41) | - |
| Slope2_CRCL_CL | - | If CrCL ≥ 100 mL/min: slope1*100+slope2(CrCL-100) | 0.00125 (8.8) | - |
| ESRD_CL | - | CL estimate for patients with ESRD | - | 0.0678 (8.3) |
| DIAL_CL | - | CL estimate for patients on dialysis | - | 20.8 (9.6) |
| POW_CRCL_CL | - | If CrCL < 80 mL/min: power effect on CL | - | 1.05 (2.4) |
| LIN_CRCL_CL | - | If CrCL ≥ 80 mL/min: linear effect on CL | - | 0.00279 (3.7) |
| ARC_CL | - | Scaling factor for CrCL in subjects with ARC | - | 0.992 (17.4) |
| cIAI_CL | - | Population effect of cIAI on CL | 1.16 (2.2) | 0.406 (23.2) |
| NP_CL | - | Population effect of NP on CL | 0.999 (2.4) | - |
| ASN_CL | - | Race effect of ASN on CL | -0.161 (11.8) | -0.0865 (20.2) |
| RACE_CL | - | Race effect on CL | -0.0855 (27.0) | - |
| APACHE_CL | - | Population effect of APACHE-II on CL | - | -0.197 (8.7) |
| cUTI_V1 | - | Population effect of cUTI on V1 | 1.03 (11.1) | 0.434 (24.0) |
| cIAI_PH2_V1 | - | Population effect of cIAI phase II on V1 | - | 1.92 (25.4) |
| cIAI_PH3_V1 | - | Population effect of cIAI phase III on V1 | - | 0.329 (28.6) |
| cIAI_NP_V1 | - | Population effect of cIAI and NP on V1 | 1.14 (9.9) | - |
| cUTI_acute_V1 | - | Population effect of cUTI and acute pyelonephritis on V1 | -0.185 (41.2) | - |
| ASN_CHN_JPN_V1 | - | Race effect of ASN, CHN and JPN on V1 | -0.27 (18.6) | - |
| WT_V1 | - | Weight effect on V1 | 1.01 (12.6) | 1.08 (7.8) |
| NPv_V1 | - | Population effect of NPv on V1 | 0.297 (45.4) | 0.175 (53.3) |
| BSV on CL | - | Between-subject variability on CL | 11.4* (3.3) | 7.29* (2.0) |
| BSV on V1 | - | Between-subject variability on V1 | 31.2* (10.2) | 28.1* (6.0) |
| BSV on Q | - | Between-subject variability on Q | 27.5* (15.5) | 14.2* (8.1) |
| BSV on V2 | - | Between-subject variability on V2 | 17.5* (8.8) | 13.5* (7.0) |
| PropRUV_PH1_ | - | Proportional error phase I (%) | 20.0 (0.5) | 17.3 (0.1) |
| AddRUV_PH1_ | - | Additive error phase I (mg/L) | 162 (7.5) | 44.6 (0.5) |
| PropRUV_PH2-3_ | - | Proportional error phase II & III (%) | 33.7 (2.1) | - |
| AddRUV_PH2-3_ | - | Additive error phase II & III (mg/L) | 4.29 (447) | - |
| PropRUV_PH2_ | - | Proportional error phase II (%) | - | 49.2 (3.0) |
| PropRUV_PH3_ | - | Proportional error phase III (%) | - | 36.3 (1.1) |

* expressed as coefficient of variation (CV%). Residual errors are expressed as standard deviations. CrCL: creatinine clearance; ESRD: end-stage renal disease; ARC: augmented renal clearance; cIAI: complicated intra-abdominal infection; NP: nosocomial pneumonia; ASN: non-Japanese, non-Chinese Asian; cUTI: complicated urinary tract infection; CHN: Chinese; JPN: Japanese; NPv: ventilated nosocomial pneumonia.

**Table S3.** Parameter estimates PKPD model 1^3^

| Parameter | Unit | Description | Estimate (RSE%) |
| --- | --- | --- | --- |
| V_AVI_ | L | Volume of distribution AVI | 1.2 (1.9) |
| V_CAZ_ | L | Volume of distribution CAZ | 0.42 (12) |
| k_b_ | /h | Binding rate constant | 0.040 (25) |
| del_50_ | h | Delay in BL degradation | 1 FIXED |
| k_BLasemax_ | /h | Maximum enzymatic degradation rate | 1.2 (12) |
| BN_50_ | log_10_ cfu/mL | Bacterial density at 50% of k_BLasemax_ | 4.5 (7.4) |
| fRES | - | Fraction in subpopulation 2 in log_10_ | -5.3 (7.9) |
| k_growth_ | /h | Bacterial growth rate | 1.4 (5.5) |
| k_death_ | /h | Natural bacterial death rate | 0.179 FIXED |
| Bmax | log_10_ cfu/mL | System carrying capacity | 9.8 (1.0) |
| Slope_AVI_ | L/mg.h | Slope AVI | 0.94 (11) |
| gamma_AVI_ | - | Hill coefficient AVI | 0.27 (8.7) |
| Slope_CAZ_ | /h | Slope CAZ | 0.024 (12) |
| gamma_CAZ_ | - | Hill coefficient CAZ | 0.64 (7.7) |
| Shift | - | Decreased susceptibility for subpop. 2 | 4.4 (2.8) |
| SYN_MAX_ | - | Maximum potentiation | 1 FIXED |
| SYN_50_ | mg/L | AVI concentration at 50% SYN_MAX_ | 0.034 (15) |
| BNI_50_ | log_10_ cfu/mL | Bacterial density at 50% Slope_AVI_ | 6.9 (0.73) |
| k_eo_ | /h | Rate constant for effect compartment | 0.47 (18) |
| RES_AVI_ | % | Proportional residual error AVI | 5.20 (9.5) |
| RES_CAZ_ | % | Proportional residual error CAZ | 26.3 (25) |
| RES_cfu_ | % | Proportional residual error cfu | 118 (6.5) |
| RRES_cfu_ | % | Proportional replicate residual error cfu | 14.1 (4.8) |

Residual errors are expressed as standard deviations.

**Table S4.** Parameter estimates PKPD model 2^4^

| Parameter | Unit | Description | Estimate (RSE%) | | |
| --- | --- | --- | --- | --- | --- |
|  |  |  | 2154 | NCTC 10783 | 9750 |
| Nmax | log_10_ cfu/mL | Maximum achievable carrying capacity in the system | 9.89 (2.1) | 9.07 (1.9) | 8.66 (1.3) |
| k_growth_ | /h | Bacterial growth rate constant associated with log_10_ of active population | 0.346 (20.9) | 0.319 (7.1) | 0.418 (6.2) |
| E_max_ | /h | Maximum kill rate constant due to CAZ | 0.240 (15.9) | 0.347 (5.6) | 0.269 (7.7) |
| A | mg/L | First parameter of bi-exponential function to characterize CAZ EC_50_ in monotherapy | 52.3 (17.2) | 149 (11.2) | 248 (9.1) |
| B | mg/L | Second parameter of bi-exponential function to characterize CAZ EC_50_ in monotherapy | 12.6 (26.0) | 33.2 (17.0) | 18.3 (14.0) |
| α | L/mg | Exponential constant associated with parameter A that describes the relationship between AVI concentration and potency of CAZ | 2.38 (119) | 0.610 (17.5) | 0.476 (3.3) |
| β | L/mg | Exponential constant associated with parameter B that describes the relationship between AVI concentration and potency of CAZ | 0.0967 (7.0) | 0.0745 (13.8) | 0.00423 (22.2) |
| γ | - | Hill coefficient that determined the steepness of the slope of the sigmoidal E_max_ curve associated with AVI enhancement of CAZ potency | 2.60 (34.2) | 4.03 (38.5) | 1.70 (6.0) |
| δ_1_ | /h | Exponential constant of the delay function to retard initial growth of the active population P1 | 0.0423 FIXED | 0.0402 (41.5) | 0.0593 FIXED |
| δ_2_ | /h | Exponential constant of the delay function to retard initial kill of the active population P1 | 0.213 (17.5) | 0.0521 (111) | 0.311 (14.2) |
| k_1-2_ | log_10_ cfu/mL/h | Rate constant for the conversion of bacterial cells from active to resting states | 0.005 FIXED | 0.005 FIXED | 0.005 FIXED |
| Deg_max_ | /h | Maximum degradation rate of CAZ | 0.0771 (51.9) | 0.417 (5.6) | 0.260 (10.0) |
| k_m_ | log_10_ cfu/mL | log_10_-transformed cfu density that yielded 50% of the maximum degradation rate | 8.5 FIXED | 8.5 FIXED | 8.5 FIXED |
| φ | - | Hill coefficient that determined the slope of the sigmoidal E_max_ model for CAZ degradation | 1.46 (82.2) | 10.1 (12.1) | 3.49 (1.0) |
| IC_50_ | mg/L | AVI concentration that yielded 50% decrease in the degradation rate | 1.96 (58.2) | 1.06 (27.1) | 2.09 (16.4) |
| AddRUV | log_10_ cfu/mL | Additive error | 0.900 | 0.879 | 0.896 |

Residual errors are expressed as standard deviations.

**Reverse translation of the CAZ dose regimen from human to mouse – used in the simulated mouse dose fractionation study**

The standard dose regimen of CAZ in patients is 2000mg every 8 hours (q8h) administered as a 2h-infusion (without renal adaptation).^5^

1. Frequency of administration
   1. Rationale: Reverse translation was based on the ratio of the CAZ elimination half-life between mice and humans.
   2. Details: The average elimination half-life for CAZ used was 0.92 hours in mice and 1.87 hours in humans, resulting in a ratio of approximately 0.5.
   3. Calculation: To reflect the 0.5 ratio in elimination half-lives between humans and mice, q8h in humans became q4h in mice.
2. Mode of administration
   1. Rationale: Continuous infusions are impractical to use in mouse studies.
   2. Details: Therefore, it was assumed that the antibiotics were administered as subcutaneous injections in mice.
   3. Simulation: The SC injections were simulated in the PK models for mice as a 0.3-h infusion.
3. Dose
   1. Rationale: Reverse translation was based on achieving equivalent CAZ-free area under the concentration-time curve over 24 hours (*f*AUC_0-24h_) in the two species.
   2. Methods: This was determined through PopPKPD simulations with n=10,000 patients administered CAZ/AVI 2000/500 mg q8h (as a 2h-infusion) for hospital-acquired pneumonia caused by *K. pneumoniae* NCTC 13438 (MIC_CAZ/AVI_ = 4 mg/L) with a starting inoculum of 6 log_10_ cfu/mL.
   3. Results: The median CAZ *f*AUC_0-24h_ in humans was approximately 650 mg·h/L.
   4. Adjustment: Using a q4h dosing interval and SC injection in the mouse model, a dose to achieve the same CAZ *f*AUC_0-24h_ was computed.
   5. Outcome: A dose of 240 mg/kg in mice was determined to match the equivalent CAZ *f*AUC_0-24h_.

**Covariate settings for CAZ/AVI PopPK models in simulated human dose fractionation study**

The following covariate settings from CAZ/AVI PopPK models were used in the simulated dose fractionation study:

- Weight: 70 kg
- Age: 65 years old
- Creatinine clearance (CrCL): 80 mL/min (indicating neither augmented renal clearance (ARC), nor end-stage renal disease (ESRD), and no dialysis)
- Patient population: Hospital-acquired pneumonia (not ventilated)
- Race: Non-Asian
- Sex: Male
- Study phase: Phase III patients
- APACHE-II score: ≤10

**Table S5.** Values for the coefficient of determination (r^2^) for the PK/PD index analysis (curve-fitting) in mouse for each bacterial strain

| Bacterial strain | PK/PD index | r^2^ value |
| --- | --- | --- |
| *K. pneumoniae* NCTC 13438 | *fT*>C_T_ | 0.790 |
|  | *f*C_max_/MIC | 0.654 |
|  | *f*AUC/MIC | **0.948** |
| *K. pneumoniae* KP981690 | *fT*>C_T_ | 0.703 |
|  | *f*C_max_/MIC | 0.574 |
|  | *f*AUC/MIC | **0.941** |
| *E. cloacae* EL871203 | *fT*>C_T_ | 0.723 |
|  | *f*C_max_/MIC | 0.652 |
|  | *f*AUC/MIC | **0.960** |
| *P. aeruginosa* NCTC 10783 | *fT*>C_T_ | **0.772** |
|  | *f*C_max_/MIC | 0.143 |
|  | *f*AUC/MIC | 0.448 |
| *P. aeruginosa* 9750 | *fT*>C_T_ | **0.701** |
|  | *f*C_max_/MIC | 0.135 |
|  | *f*AUC/MIC | 0.386 |
| *P. aeruginosa* 2154 | *fT*>C_T_ | **0.849** |
|  | *f*C_max_/MIC | 0.102 |
|  | *f*AUC/MIC | 0.482 |

In bold: the highest r^2^ per strain.

**Table S6.** Values for the coefficient of determination (r^2^) for the PK/PD index analysis (curve-fitting) in human stratified by mode of infusion for each bacterial strain

| Bacterial strain | PK/PD index | 0.5h-inf. | 2h-inf. | 4h-inf. | Cont. inf. | Pooled |
| --- | --- | --- | --- | --- | --- | --- |
| *K. pneumoniae* NCTC 13438 | *fT*>C_T_ | 0.826 | 0.764 | 0.747 | ND | 0.737 |
|  | *f*C_max_/MIC | 0.723 | 0.703 | 0.744 | 0.909 | 0.706 |
|  | *f*AUC/MIC | **0.902** | **0.886** | **0.894** | **0.918** | **0.902** |
| *K. pneumoniae* KP981690 | *fT*>C_T_ | 0.403 | 0.329 | 0.321 | ND | 0.313 |
|  | *f*C_max_/MIC | 0.593 | 0.527 | 0.565 | 0.727 | 0.578 |
|  | *f*AUC/MIC | **0.727** | **0.688** | **0.710** | **0.744** | **0.716** |
| *E. cloacae* EL871203 | *fT*>C_T_ | 0.904 | 0.869 | 0.863 | ND | 0.860 |
|  | *f*C_max_/MIC | 0.738 | 0.748 | 0.789 | 0.935 | 0.730 |
|  | *f*AUC/MIC | **0.928** | **0.917** | **0.924** | **0.943** | **0.928** |
| *P. aeruginosa* NCTC 10783 | *fT*>C_T_ | **0.719** | **0.697** | **0.720** | ND | 0.699 |
|  | *f*C_max_/MIC | 0.388 | 0.436 | 0.478 | **0.905** | 0.446 |
|  | *f*AUC/MIC | 0.680 | 0.691 | 0.696 | **0.904** | **0.733** |
| *P. aeruginosa* 9750 | *fT*>C_T_ | **0.675** | **0.680** | **0.706** | ND | 0.678 |
|  | *f*C_max_/MIC | 0.333 | 0.383 | 0.421 | **0.905** | 0.397 |
|  | *f*AUC/MIC | 0.629 | 0.640 | 0.643 | **0.903** | **0.689** |
| *P. aeruginosa* 2154 | *fT*>C_T_ | **0.698** | **0.695** | **0.686** | ND | **0.678** |
|  | *f*C_max_/MIC | 0.381 | 0.362 | 0.404 | **0.807** | 0.396 |
|  | *f*AUC/MIC | 0.608 | 0.590 | 0.598 | **0.806** | 0.635 |

ND: could not determinate r^2^ value due to the impossibility of fitting an EMAX model to the data. In bold: the highest r^2^ per strain and infusion mode.

**References**

1. Sy SKB, Zhuang L, Xia H, Schuck VJ, Nichols WW, Derendorf H. A model-based analysis of pharmacokinetic–pharmacodynamic (PK/PD) indices of avibactam against Pseudomonas aeruginosa. *Clinical Microbiology and Infection* 2019; **25**: 904.e9-904.e16.

2. Li J, Lovern M, Green ML, *et al.* Ceftazidime‐Avibactam Population Pharmacokinetic Modeling and Pharmacodynamic Target Attainment Across Adult Indications and Patient Subgroups. *Clin Transl Sci* 2019; **12**: 151–63.

3. Kristoffersson AN, Bissantz C, Okujava R, *et al.* A novel mechanism-based pharmacokinetic–pharmacodynamic (PKPD) model describing ceftazidime/avibactam efficacy against β-lactamase-producing Gram-negative bacteria. *Journal of Antimicrobial Chemotherapy* 2020; **75**: 400–8.

4. Sy SKB, Zhuang L, Xia H, *et al.* A mathematical model-based analysis of the time–kill kinetics of ceftazidime/avibactam against Pseudomonas aeruginosa. *Journal of Antimicrobial Chemotherapy* 2018; **73**: 1295–304.

5. European Medicines Agency. Fortum (ceftazidime): Summary of product characteristics. Available at: https://www.ema.europa.eu/en/documents/referral/fortum-article-30-referral-annex-iii_en.pdf.
